# A new direction in managing avulsed teeth: stem cell-based de novo PDL regeneration

**DOI:** 10.1186/s13287-022-02700-x

**Published:** 2022-01-28

**Authors:** Hacer Aksel, Xiaofei Zhu, Philippe Gauthier, Wenjing Zhang, Adham A. Azim, George T.-J. Huang

**Affiliations:** 1 Department of Bioscience Research, College of Dentistry, University of Tennessee Health Science Center, Memphis, TN USA; 2 Department of Periodontics and Endodontics, School of Dental Medicine, University at Buffalo, Buffalo, USA; 3 Department of Endodontics, Henry M. Goldman School of Dental Medicine, Boston University, Boston, MA USA; 4 VIP Dental Service and Geriatric Dentistry, Peking University School and Hospital of Stomatology, Beijing, China; 5 Département d’endodontie, Faculté de Médecine Dentaire, Université Laval, Québec, QC G1V 0A6 Canada; 6 Department of Genetics, Genomics and Informatics, College of Medicine, University of Tennessee Health Science Center, Memphis, USA; 7 Department of Endodontics, Arthur A Dugoni School of Dental Medicine, University of Pacific, San Francisco, California USA; 8 Cancer Research Building, University of Tennessee Health Science Center, 19 S. Manassas St. Lab Rm 256, office 255, Memphis, TN 38163 USA

**Keywords:** Avulsion, Traumatic injury, Periodontal ligament (PDL), Periodontal ligament stem cells (PDLSCs), Cell-based PDL regeneration, Cell sheet, Tooth replantation, Animal models, Mini-swine, Dog

## Abstract

Management of avulsed teeth after replantation often leads to an unfavorable outcome. Damage to the thin and vulnerable periodontal ligament is the key reason for failure. Cell- or stem cell-based regenerative medicine has emerged in the past two decades as a promising clinical treatment modality to improve treatment outcomes. This concept has also been tested for the management of avulsed teeth in animal models. This review focuses on the discussion of limitation of current management protocols for avulsed teeth, cell-based therapy for periodontal ligament (PDL) regeneration in small and large animals, the challenges of de novo regeneration of PDL on denuded root in the edentulous region using a mini-swine model, and establishing a prospective new clinical protocol to manage avulsed teeth based on the current progress of cell-based PDL regeneration studies.

**Supplementary Information:**

The online version contains supplementary material available at 10.1186/s13287-022-02700-x.

## Background

In the field of traumatic dental injury, especially the management of avulsed teeth, it has been a lack of significant breakthrough in the past many decades. Avulsion is the most damaging injury to the periodontal ligament (PDL). The timing of replantation is the most critical determining factor for the outcomes. Protocols have been proposed using various storage media for an avulsed tooth before replantation, such as Hanks’ balanced salt solution (HBSS) [1] or propolis [2] to improve the survival of PDL cells. However, besides identifying the best tooth storage media, which is often not available at the site of an avulsion, there has been little advancement to treat this condition.

The complete absence of PDL leads to direct contact of bone with the cementum, and if more than 20% of the PDL is injured, an abnormal attachment can occur after healing takes place [3]. After initial inflammation, a diffuse area of the root surface is devoid of cementum. Surrounding PDL cells will compete to repopulate it, and often bone precursor cells will move across from the socket wall and populate the damaged root rather than slower-moving PDL cells. This leads to ankylosis, and the root will eventually be replaced by bone [4]. This process may affect the growth of the alveolar ridge and can compromise function and esthetics. No treatment is really effective to prevent ankylosis.

Stem cell-mediated regeneration therapy emerged in the 1990s as part of the modern tissue engineering concept. The introduction of such a concept into PDL regeneration appears the most promising direction for salvaging avulsed teeth. Utilizing periodontal ligament stem cells (PDLSCs) to regenerate PDL could theoretically prevent or lower the incidence of resorption following tooth replantation, especially when there is extended extraoral time. The question is, how do we apply PDLSCs onto the root surface such that PDL can be completely regenerated and perform normal functions? Early in vivo studies have indicated the potential use of PDLSCs to regenerate PDL-like tissue using small animal and relatively simple models [5, 6]. Large animal models have also been used to test proof-of-principle periodontal tissue regeneration for restoring periodontal defects from periodontal diseases [7–9]. Large animal models were subsequently tested for PDL regeneration of the avulsed teeth, demonstrating a breakthrough for the management of avulsed teeth [10–12].

Emerging stem cell banking system could serve as a source of autologous or allogeneic stem cells for clinical use soon after the trauma has taken place. If the autologous cell source is not available, genotyped allogeneic cell source may be used when the stem cell banking system is in place.

This review article focuses on the significant breakthrough, albeit a small step on the stem cell-based PDL regeneration of avulsed teeth. We discuss the challenges we encountered with the large animal models tested for de novo regeneration of PDL for avulsed teeth. Finally, we propose a protocol for managing avulsed teeth with a stem-cell-mediated approach.

## The outcome of PDL damage from traumatic injury

Avulsion injury mostly occurs in children and young adults. It is a common dental injury involving total displacement of a tooth out of its alveolar socket which accounts for 0.5 to 16% of dental injuries [13, 14]. The prognosis depends on the measures taken at the time and place of accident, type of medium storage, and the time immediately after the avulsion with immediate replantation being the treatment of choice for these patients. The occurrence of replacement resorption increased with extended periods of dry storage. Replacement resorption ranges from only 9.5% in teeth with short dry storage of below 15 min to 100% in teeth with dry storage exceeding 60 min [13, 15–17].

If ankylosis occurs after replantation, it often affects the growth of the alveolar ridge and the eruption and position of the adjacent teeth [18, 19]. Function and esthetics could be compromised in these patients, making them difficult for the dental practitioner to treat successfully and predictably. A study of replantation in 400 traumatically avulsed permanent incisors showed total success in only 36% after replantation [16]. A recent retrospective clinical study reported outcomes from 36 patients and 49 replanted permanent teeth within a 3.5 year-observation period [20]. Replacement resorption was observed in 51% of the teeth (25/49), while only 26.5% (13/49) of the teeth showed functional healing, indicating the importance of maintaining or regenerating PDL after tooth avulsion.

Damage to the PDL as a result of avulsion cannot be avoided. If the PDL left attached to the root surface does not dry out, the consequences of tooth avulsion are usually minimal [21]. Numerous studies have tried to evaluate ways to improve the prognosis of tooth avulsion using various tooth storage media. Suggested storage media in order of preference are milk, saliva either in the vestibule of the mouth or in a container, and water being the worst due to its hypotonicity which causes rapid PDL cell death [1, 22]. HBSS also has been proven to maintain the viability of PDL cells for an extended time [1]. Propolis was found more effective than HBSS in maintaining PDL cell viability [2]. However, the limitation with storage medium generally is that it has to be present at the time of injury to be effective. The medium also has to be stored at recommended optimal temperatures. Various agents (acidic and alkaline solutions, antibiotics, corticosteroids, enamel matrix protein, and fluoride) have been investigated for root conditioning prior to replantation to improve cellular viability and attachment while decreasing inflammation. However, none of the methods are predictable to prevent tooth ankylosis or inflammatory resorption in severely damaged PDL [23].

Extraoral dry time reduces PDL cell viability and extracellular matrix (ECM) protein integrity increasing inflammation and root resorption. A previous study reported changes in the proteomic profiles in human PDL after delayed or dry preservation of the tooth. There was a gradual reduction in vimentin concentration from immediate to 2-week storage at 4 °C and in dry conditions [24]. Understanding the functions and roles of ECM proteins for PDL regeneration can help design future regenerative approaches [25–27]. Another study showed that after 30 min of dry time, an increased expression of proinflammatory marker and matrix metalloproteinases (MMPs)-3 and MMP-9 was observed compared to 15-min dry time [28]. Even 20 min of extraoral dry time was shown to have a detrimental effect on PDL cell viability and PDL attachment, causing replacement resorption [29]. Emdogain was also ineffective in preventing the onset or progression of the root resorption after 20 min of extraoral dry time [29].

## Cell-based PDL regeneration for periodontal defects

The initial application for cell-based periodontal therapy was to repair periodontal defects due to periodontal diseases. Various in vitro and in vivo experimental models have been used to demonstrate the cell-based regeneration of periodontal tissues.

### Cell-based PDL regeneration potential in small animal models

After PDLSCs were discovered and shown to regenerate ectopic PDL-like and cementum-like tissues using hydroxyapatite/tricalcium phosphate (HA/TCP) model in immunocompromised mice [30], they were tested to determine whether they can regenerate PDL and cementum on the root surface. This was first tested using a cell-sheet technology. PDL cell sheets placed on a dentin block and implanted subcutaneously in athymic rats showed significantly more cementum-like tissue and structures resembling Sharpey’s fibers [6]. Hasegawa et al. transplanted cell sheet from human PDLCs into a dehiscence model in athymic rats and reported cementum and PDL regeneration, while no tissue formation was observed in the control group suggesting cell sheet engineering as a promising approach for PDL regeneration [31]. In a recent study, a complex cell sheet was fabricated using rat PDL cells and osteoblasts. Ectopic and orthotopic transplantation of the construct in vivo for 8 weeks showed three-dimensional bone-ligament regeneration around the tooth surface [32]. Similarly, the combinational use of biphasic scaffold with cell sheet technology that had a bone compartment coated with calcium phosphate and osteoblasts and periodontal compartment consisting of PDL cell sheet was shown to provide simultaneous formation of alveolar bone, PDL and cementum after subcutaneous implantation in rats [33].

### Cell-based PDL regeneration for periodontal defects in large animal models

Although the rodent model is relatively less costly to study, their size of teeth and their overall biological difference from humans are substantial. Thus, large animal as a study model is a necessary step to acquire preclinical information. A canine model showed PDL and cementum regeneration when the PDL cell sheet was incubated with an osteoinductive medium prior to transplantation into periodontal defects [9].

Different types of stem/progenitor cells were also transplanted into pig models for periodontal regeneration. Human dental pulp stem cells, PDLSCs or adipose tissue-derived multi-lineage progenitor cells were used for the treatment of a large bone defects with enhanced periodontal tissue healing [34–37].

### Cell-based PDL regeneration for periodontal defects in humans

In human systems, one report showed that autologous PDL progenitor cells (PDLPs) may be used to treat periodontally involved teeth with deep intrabony defects [38]. Three patients were involved in the study. The diseased roots were treated by directly transplanting a construct of PDLCs and bone graft into the bony defect. After a 72-month follow-up, all patients showed improved periodontal health compared to presurgical conditions.

Autologous PDLSCs have also been used in clinical trials for the treatment of periodontal defects. Chen et al. conducted a randomized clinical trial to use autologous PDLSC sheet as an adjunctive grafting material in guided tissue regeneration of 20 teeth and did not find a significant difference for the alveolar bone height compared to the control teeth receiving only graft material [39]. In another study, Iwata et al. used ex vivo cultured autologous and three-layered PDL cell sheet in the treatment of severe periodontal defects in 10 patients. No adverse effects had been reported during 55 ± 19 months. Reduction of the probing depth, clinical attachment gain and increase in radiographic bone level were reported at 6 months after the transplantation [40].

## Cell-based PDL regeneration for avulsed teeth

A recent systematic review investigated the outcomes of cell-based approaches for the tooth replantation using animal models and reported that the replacement root resorption and ankylosis ranged from 20.8 to 100% in the cell-free control group and 0 to 38.4% in the cell-based groups suggesting the potential of cell-based approach for the management of avulsed teeth [41].

Strategically, two key issues should be considered when implementing cell-based PDL regeneration therapy for avulsed teeth: (i) access to stem cells when needed through stem cells banking systems as an essential step; (ii) develop a more systematic protocol to achieve a clinically feasible regimen. A streamlined and efficient process to regenerate PDL for avulsed teeth should be established.

Regarding the type of stem cells, PDLSCs have been shown to have great potential for regenerating PDL, cementum and repairing periodontal bone defect [9, 30]. If tissue regeneration technologies can repair PDL, the replacement resorption should not occur after replantation. Therefore, using stem cell-based regeneration of PDL on avulsed teeth may revolutionize how we manage these cases. While specific and advanced technology is still lacking in these studies, they provide a direction as to what the next step of managing avulsed teeth should be heading. We will overview these studies and their findings in the following.

### Cell-based PDL regeneration for avulsed teeth in small animal models—rat

Using athymic rat molar as a study model, Dangaria et al. seeded mouse PDL progenitor cells onto extracted maxillary molars that have been denuded of soft tissues and replanted back to the socket 4 days after the extraction. They found that PDL was completely regenerated, whereas the control group without cells had resorption of ankylosis or lost (Table 1) [42].

**Table 1:** Animal studies for cell-based PDL regeneration of avulsed teeth

References	Animal	Tooth type	RCT	Tooth out time	Cells	Experiment groups	Key method	Time point studied	Outcome						
Dangaria et al. [42]	Rat	Molars	None	4 d	Xenogenic PDLPCs	I. No cell	Cells seeded onto entire root, 3 d incubation	6 months	I. Lost, resorbed or ankylosed						
						II. Seeded m PDL pro cells			II. PDL fully formed						
Demirel et al. [43]	Rat	Incisor	RCT before replant	1 h air dry	AdSCs (litter mates)	I. Control no cells		2 months	Level of new PDL regeneration: III(92.0)>II(78.0)>I(63.9) Level of Ankylosis: I(20.8)>II(15.6)>III(7.7) Level of Inflammatory resorption: I(13.5)>II(5.0)>III(0)						
						II. Fibrin sealant	II. Fibrin sealant into sock before replant								
						III. ATSCs + Fibrin sealant	III. ATSCs + Fibrin sealant into socket before replant								
Zhou et al. [11]	Dog	Premolar	RCT before replant	2 d	Auto PDL Fbs,	I. Control no cells II. Cell sheet	Multi-layer cell sheet, wrapped onto root, replant back to same socket	8 wks	Cell sheet group had ~9x better PDL healing and ~8-fold less replacement resorption						
Zhao et al. [10]	Dog	Incisors	2 wks after replant	2 h dry	Auto PDLSCs	I. Cell sheet/PRF II. Cell sheet III. PRF IV. No graft (control)	Cell sheet and or PRF cut into fragments and filled into socket before replant	8 wks	Level of new PDL regeneration: I(45.8)*>II(35.9)>III(30.4)>IV(12.5) Level of replacement resorption: IV(30.4)>III(16.1)>II(14.1)>I(6.9) Level of surface resorption: III(51.8)>I(47.2)>IV(39.3)>II(39.1) Level of inflammatory resorption : IV(17.9)>II(10.9)>III(1.8)>I(0)						
Lee et al. [12]	Dog	Premolar	RCT before replant	5 d	Auto PDLSCs	I. Control no cells	Root surface coating with Fn and/or CaP	8 wks		PDL space	Ankylosis	w/o root resorp	w root resop	Resorp + Ankylosis	
						II. Fn coating + cells	Cells seeded onto entire root, 60 h incubation		PDLSC(+):	60.9*	39.1	38.9	22.7	38.4	
						III. CaP coating + cells			PDLSC(-):	33.7	66.3	-	-	100	
						IV. CaP/Fn coating + cells									

Fbs: fibroblasts. m PDL pro: mouse PDL progenitor. AdSC: adipose tissue-derived stem cells. RCT: root canal treatment. Wks: weeks. Auto: autologous. PRF: platelet-rich fibrin. Fn: fibronectin. CaP: calcium phosphate

*%

A report testing PDL regeneration for avulsed teeth was conducted in rats using their maxillary incisors. Adipose tissue-derived stem cells (AdSCs) were used as a cell source [43]. The study applied fibrin sealant as a carrier for AdSCs into the tooth socket before replanting the tooth back. The tooth received retrograde root canal treatment and apex sealed and was let air dry for 1 h before replantation. The authors found that the AdSC group had significantly more regenerated PDL than the control group (Table 1).

### Cell-based PDL regeneration for avulsed teeth in large animal models—dog

Using cell-based approaches, a significant breakthrough in rescuing avulsed teeth was made in experiments performed in a dog model. Either fibroblasts from PDL or PDLSCs were applied onto the root or placed into the tooth socket before replantation [10–12]. To enhance regeneration, the addition of platelet-rich fibrin (PRF) or special coating of the root surface like fibronectin to augment cell attachment was tested [10]. Table 1 lists and summarizes the reports from three research teams using the dog model to test PDL regeneration for avulsed teeth with various methods.

Zhou et al. [11] used autologous PDL fibroblasts and stimulated with vitamin C for 5 days to induce extracellular matrix formation. The extracted tooth had the soft tissue removed, root canal treatment performed, and the formed cell sheet was then wrapped onto the tooth root before replanting into the same socket. Total time that the tooth was out was 2 days. Their histological evaluation of the outcome at 8 weeks showed a dramatically better PDL regeneration in the cell-sheet group and far less replacement resorption than the control group. This report is the first to confirm the need for cell-based therapy for a favorable PDL regeneration of the avulsed tooth.

Zhao et al. [10] utilized autologous PDLSCs and tested the benefit of PRF. Formed PDLSC-sheet and the collected PRF were chopped into small pieces and filled into the tooth socket up to 2 mm below the gingiva line. Extracted tooth was gently cleaned and let dry for 2 h before replantation into the cell/PRF-filled original socket. The root canal treatment was performed 2 weeks later. The outcome showed that the combination of cell-sheet and PRF fragments had better PDL regeneration with less replacement resorption compared to using cell sheet or PRF only. Transplanted PDLSCs were labeled; therefore, they could be tracked in the tissues. The majority of the cells in the regenerated PDL were from the transplanted population. This again validates the importance of cell-based therapy for avulsed teeth.

Lee et al. [12] were more concerned about the attachment of cells onto the tooth root surface before replantation. Extracted teeth received root canal treatment and were coated with fibronectin and/or calcium phosphate, followed by seeding autologous PDLSCs onto the entire root surface and underwent 60 h of incubation to allow cell growth and attachment. On the fifth day after the tooth extraction and treatment, it was replanted back to the same socket. The group using PDLSCs again showed better outcomes in PDL regeneration and less ankylosis plus resorption than the without PDLSCs.

These cell-based techniques, although preliminary, proved that avulsed teeth may be better salvaged via cell-based than cell-free approach and is a significant breakthrough for the management and rescuing of avulsed teeth.

## De novo PDL regeneration for completely denuded root implanted to edentulous region

The above-mentioned dog model studies focused on the replantation of the tooth back to the original socket. The time the tooth was out of the socket ranged from 2 h to 5 days in the experimental design. Original cells on the root surface are unlikely to survive, especially in the studies by Lee et al. [12] in which teeth were sterilized—12% ethylene oxide gas at 54 °C for 6 h and then degassed for 12 h, followed by 5% EDTA treatment. The PDL tissue that remained in the tooth socket seems well preserved if only after 2 h of extraction. These findings may explain why Ji et al. [44] had observed that a tooth replanted into a socket already healed for 3 months did not yield a good PDL regeneration using cell-free approaches. Therefore, when replanting the tooth back to the original socket within a few hours, it is likely that the survived PDL cells in the socket will play an important role in the PDL regeneration after tooth replantation. Thus, without the presence of PDLSCs, it would be tremendously challenging to achieve de novo PDL regeneration with denuded roots. One important point is that when avulsed teeth are heavily contaminated, it can be difficult to decontaminate without denuding. Replantation in such a condition will lead to infection and inflammation causing inflammatory root and adjacent bone resorption. Eventual tooth loss is unavoidable. Thus, denuding the root to decontaminate followed by cell-based de novo PDL regeneration may offer a better chance to save the tooth.

### Cell-sheet method

To overcome the difficulty of lacking in situ PDLSCs, cell sheet engineering with a temperature-responsive culture dish has been supposed to have many advantages over the methods using artificial scaffolds. Especially, the cells can be harvested as a single sheet without destroying the cellular attachment proteins and extracellular matrix [45]. Iwata et al. used this cell sheet technique to achieve periodontal regeneration of teeth having alveolar bone loss in both canine model [9] and clinical cases [46]. The cell sheet technique involves using a culture dish coated with poly N-isopropylacrylamide (PIPAAm)—a thermo-responsive polymer (UpCell^tm^; CellSeed Inc., Tokyo, Japan), such that it allows easy detachment of PDLSC sheet from the dish while attaching onto a biodegradable membrane. Cells grown in the UpCell dish detach from the surface of the dish at room temperature while staying as one layer without losing the extracellular matrix. This sheet is then collected by placing a membrane on top of the cells. Cell sheets from multiple dishes can be attached onto the same membrane. We used this cell-sheet method to test de novo PDL regeneration in both the ectopic SCID mouse model and the orthotopic swine model (Additional file 1: Fig. S1), as discussed below.

### De novo PDL regeneration for denuded root—SCID mouse subcutaneous model

Ectopic PDL regeneration using a mouse model is usually the first step of in vivo studies. Before using the root fragment, PDL/cementum regeneration potential of PDLSCs from humans or mini-swine was tested using an HA/TCP model for ectopic PDL-cementum complex formation in mice [47, 48]. Multipotent swine PDLSCs indicated in Additional file 2: Fig. S2A were supposed to differentiate toward osteogenic lineages when mixed with osteoinductive HA/TCP granules [49]. Studies from our group [47, 48] also confirmed that PDLSCs could differentiate into cementoblast-like cells which produce mineral tissues on the surface of HA/TCP granules. As shown in Additional file 2: Fig. S2B, cementum- and PDL-like tissues were formed. Human PDLSCs appeared to form some Sharpey’s fiber-like or collagen bundles in PDL-like tissues, whereas such structures were less observable in swine PDLSC regenerated tissues.

**Fig. 1:**
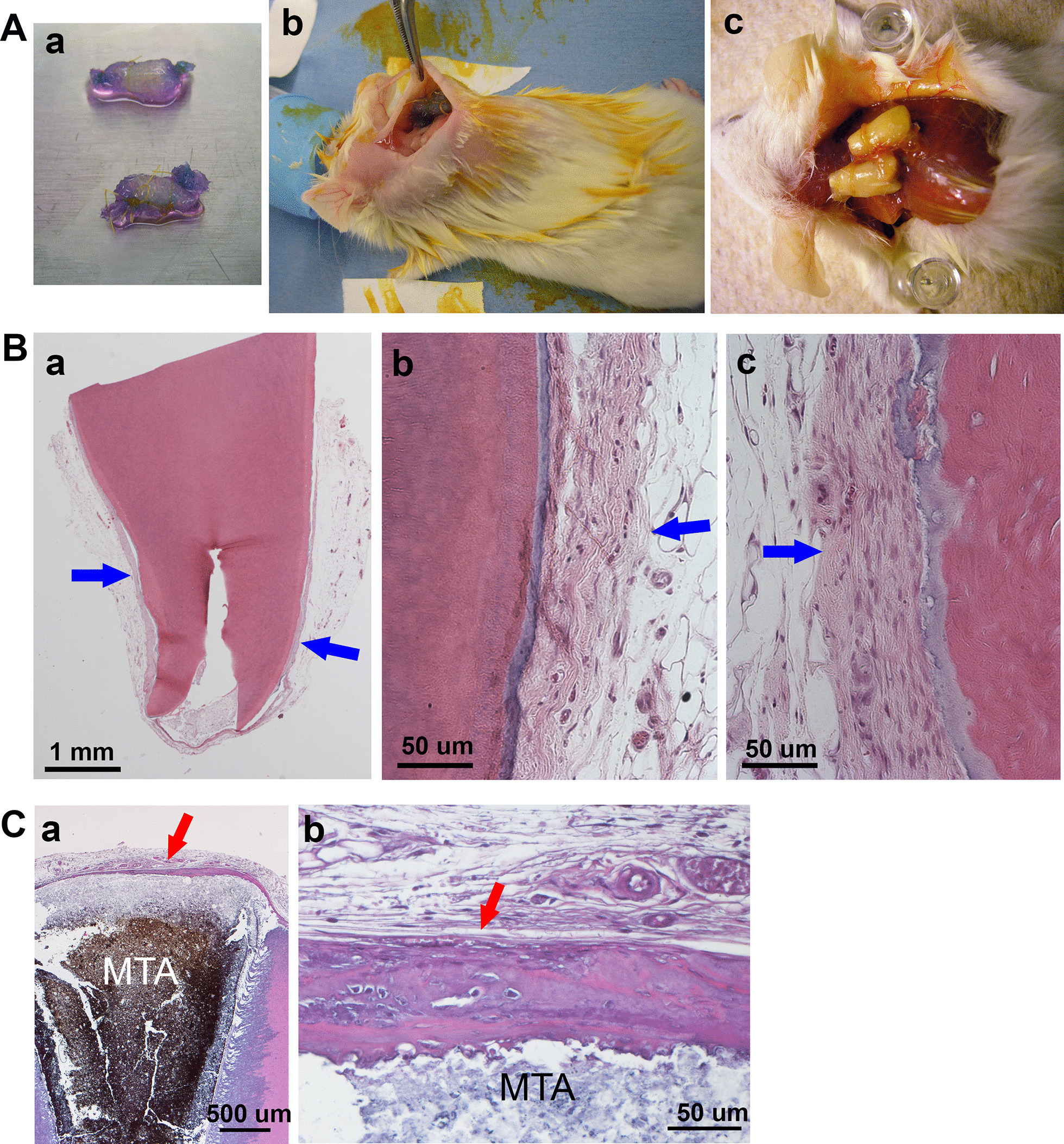
Root fragment mouse model for human PDL regeneration. **A** Root fragment transplantation into SCID. (Aa) A root fragment wrapped with PGA/PDLSC sheets. Cells were treated with vitamin C for one week as reported previously [47]; (Ab) fragments inserted into dorsal subcutaneous space of SCID mice; (Ac) root fragments retrieved ~ 3 months later and decalcified/processed for histology. **B**, **C** H&E histology of root fragments. (Ba) Root encapsulated by a layer of soft connective tissue; (Bb,c) magnified views of the PDL-like tissues. (Ca) A root fragment coronal end showing MTA (mineral trioxide aggregate) cement seal of the canal opening. The MTA is covered by a layer of mineral tissue; (Cb) magnified views of cementum-like tissue. No cementum-like tissue was observed on the root surface. Blue arrows: PDL-like tissue; red arrows: calcified cementum-like

For simulation of PDL regeneration after tooth avulsion, we tested de novo PDL regeneration from the denuded root in a SCID mouse model using the cell sheet technique (Fig. 1). Human roots deprived of cellular contents were treated with 1.5% NaOCl, followed by 17% EDTA and normal saline. Three layers of human PDLSCs grown to over-confluent in UpCell dishes were attached onto a biodegradable polyglycolic acid (PGA) membrane (Neoveil, Gunze Ltd, Japan). This cell-sheet membrane was wrapped onto the human root with cells facing the root surface and tied with resorbable sutures (Fig. 1Aa). The PGA/cells/root construct was transplanted into the dorsal subcutaneous space of SCID mice and later harvested (Fig. 1Ab,c). We observed a layer of PDL-like tissue surrounding the root, however having minimal to no cementum regeneration on the root surface (Fig. 1B, C).

### De novo PDL regeneration for denuded root into edentulous region—orthotopic mini-swine model

**Fig. 2:**
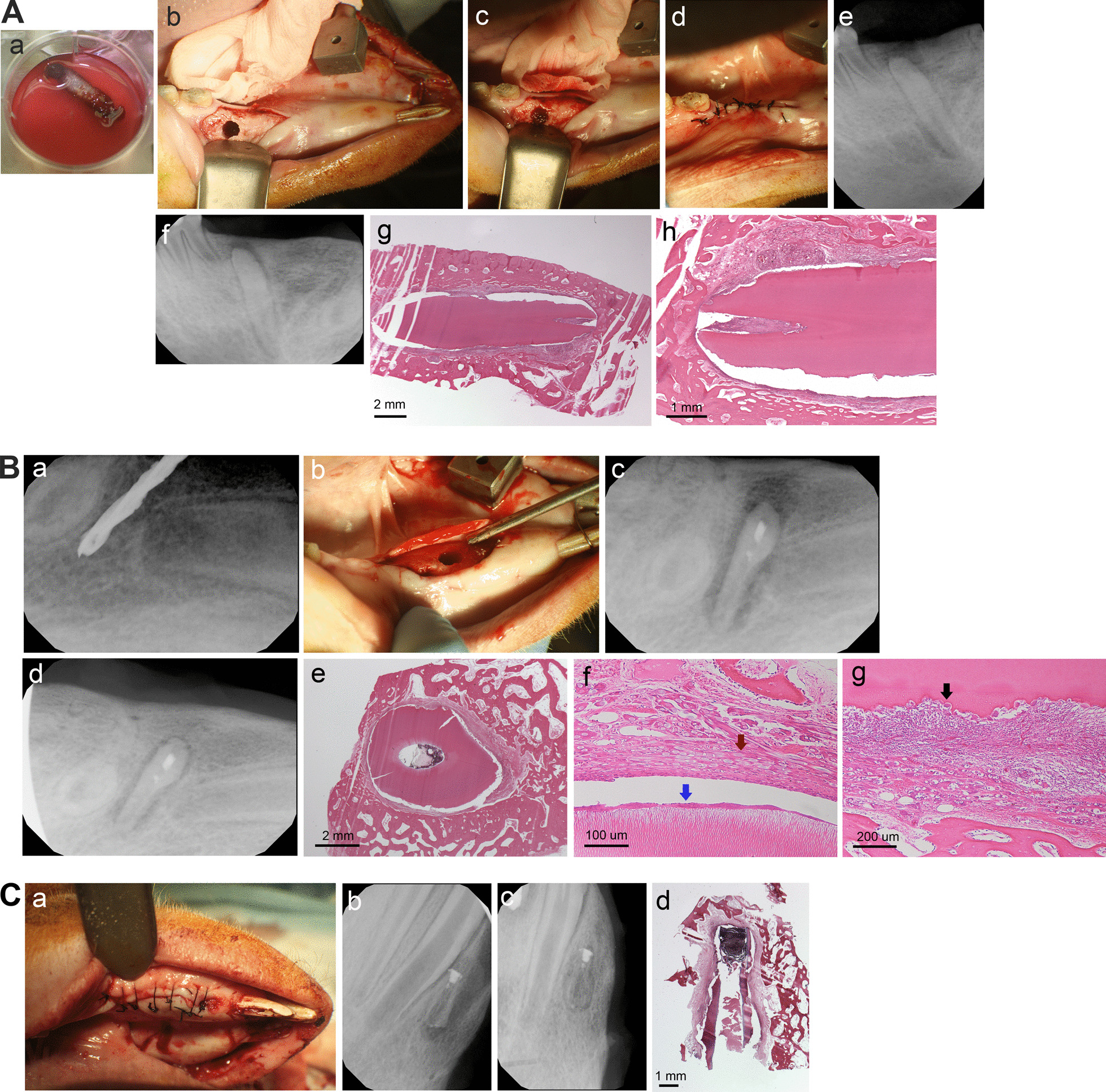
Orthotopic PDL regeneration for swine roots. Swine roots with allogeneic PDLSC/GMSC sheets were inserted into the mandibular jawbone using implant drills for osteotomy. **A** PDLSC-sheet/root implantation. (Aa-d) Cell sheet wrapped on the root was inserted into the created socket in the mandibular edentulous area and the wound sutured; (Ae) radiograph right after root insertion; (Af) radiograph ~ 8 weeks later at sacrifice; (Ag,h) H&E histology of the inserted root showing resorption and inflammation around the root. **B** GMSC sheets/root implantation. (Ba-c) Implant drill osteotomy and insertion of the root wrapped with GMSC sheets; (Bd) radiograph at sacrifice ~ 6 weeks later; (Be-g) H&E histology showing resorption and inflammation. Blue arrow in (Bf) showing possible newly deposited cementum; red arrow in (Bf) showing remaining PGA membrane fibers; black arrow in (Bg) showing osteoclasts resorbing root surface. **C** PDLSC-sheet/root implantation. (Ca,b) A root wrapped with PDLSC sheets was inserted into the mandibular anterior teeth region; (Cc) radiograph at sacrifice 3 months later showing root resorption; (Cd) H&E histology showing root dentin severe resorption. Cells were treated with vitamin C for one week as reported previously [47]. GMSC: gingival mesenchymal stem cells.

**Fig. 3:**
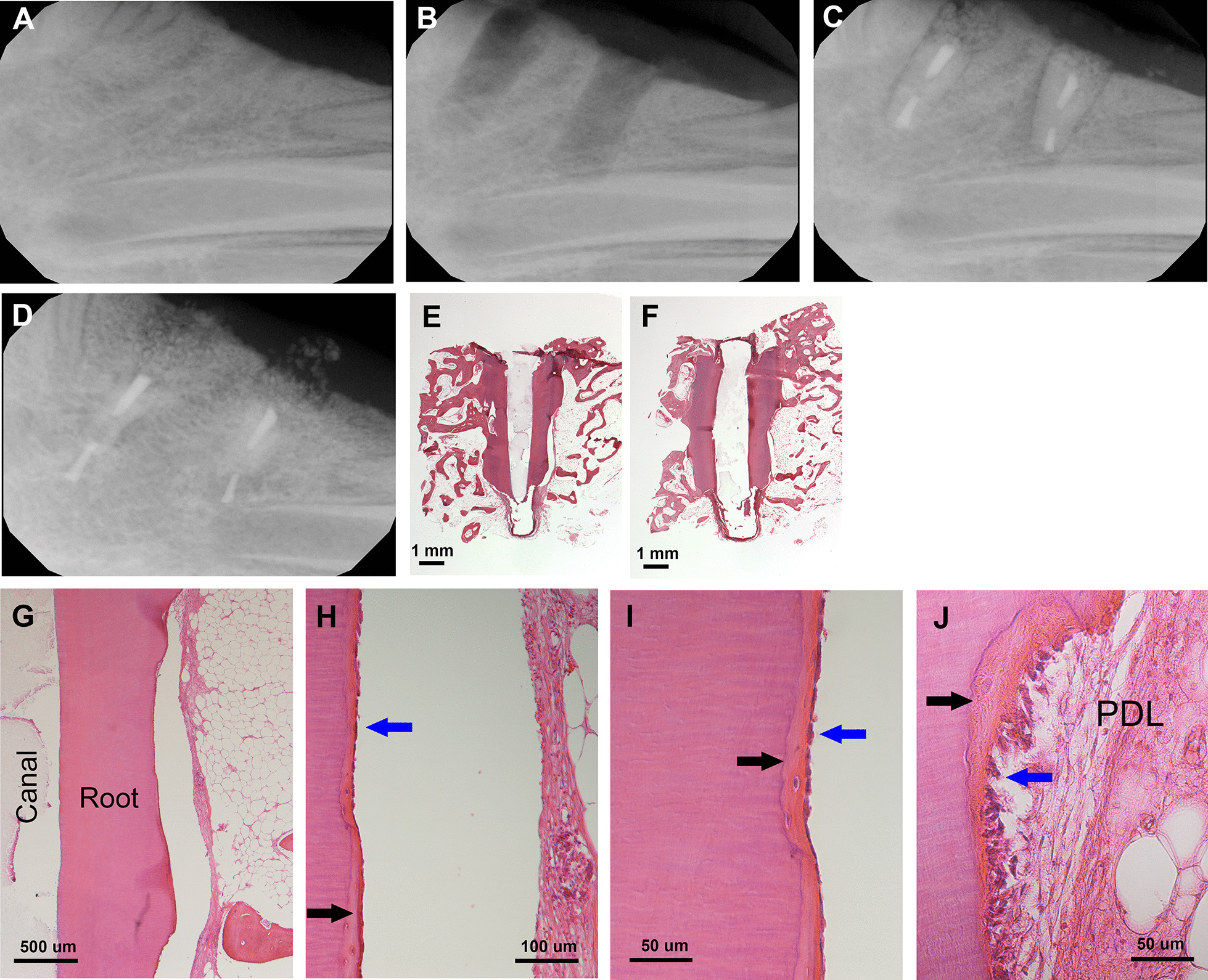
Orthotopic PDL regeneration for swine roots with autologous PDLSC sheets. **A** Mandibular edentulous region. **B** Osteotomy was performed by implant drills to create insertion space. **C** Radiograph showing two root fragments were inserted into the space and the wound sutured. **D** radiograph ~ 4 months later at sacrifice. **E**, **F** H&E histology of the inserted root showing resorption of the roots. **G**–**J** Histology showing root dentin resorption. Black arrows: newly deposited cementum-like; blue arrows: cementoblast-like cells. Cells were treated with vitamin C for one week as reported previously [47]

Swine teeth were treated and prepared as mentioned above in the mouse model. An alveolar bone socket was created by dental implant drills in the edentulous region of the swine mandible (Additional file 3: Fig. S3 and Figs. 2 and 3). The root wrapped with three layers of swine PDLSC sheets on the PGA membrane tied with sutures was transplanted into the created socket and the wound closed. At ~ 3–4 months, the teeth were processed for histological analysis after animals were euthanized. The implanted roots tended to be extruded out of the mandible and lost (Additional file 3: Fig. S3) or resorbed (Figs. 2 and 3) in the bone over time based on radiographic observation. Histologically, there were soft connective fibrous tissue surrounding the root with inflammatory infiltrates and root surface resorption. Although some newly deposited cementum-like tissue could be detected (Fig. 2B–f; Fig. 3H–J), the overall finding of the histological sections is inflammation and resorption.

### Problems using cell-sheet/membrane method for de novo regeneration of PDL for the entire root

Several possibilities may have attributed to the lack of PDL regeneration along with root resorption and inflammation using this approach: i) Using cell sheet attached to membrane method may not have allowed cells to migrate onto root surface as anticipated. This is witnessed in both the mouse subcutaneous model and swine orthotopic model. Inserting the root into the socket may cause shifting of the cell/membrane preventing cell attachment to the root. ii) Drilling a socket in the edentulous region may have caused a greater inflammatory response from the trauma. Along with a large foreign material, membrane was entering the host, which may have intensified inflammatory response damaging transplanted cells before inflammation subsides.

## Functional aspects of PDL regeneration

### Topographic alignment and nonuniformity of PDL fibers

The complex fibrillar structure of PDL is challenging as the regenerated PDL needs to withstand the occlusal forces. The spatial orientation of PDL fibers is essential for its function during mastication [50]. PDL cells can replace and regenerate collagen fibers. However, the collagen density in the PDL is not uniform with a denser network in the collar region and more mature collagen fibers on the cementum side compared to the bone side that is correlated with the biomechanical properties of the PDL tissue [51]. PDL space is very narrow (150–400 µm) limiting the placement of scaffold materials and keeping the material between two hard tissues is also challenging during PDL regeneration [50].

### Mechanical stimulation and traumatic occlusion

Mechanical stimulation is essential for the extracellular matrix production, alignment of collagen fibers for the mechanical properties and functions of the PDL [52–54]. On the other hand, traumatic occlusion interferes with the cell viability, fiber alignment, orientation and healing of the PDL. Studies have shown that traumatic occlusion increased PDL width and bone resorption, and reduced bone area by delaying repair [55, 56]. Mechanical loading of PDL is essential to preserve the PDL space, improve the regeneration and prevent ankylosis and calcification of the fibers [57, 58]. Conversely, mechanical stability is essential, and excessive loading should be avoided to allow normal PDL tissue regeneration [50].

### Multiple aligned tissue formation

PDL tissue regeneration compromises the hard and soft tissue regeneration concurrently. It requires the recruitment and differentiation of cells to form a spatial organization of multiple tissues, PDL, cementum and alveolar bone. PDLCs can differentiate into PDL fibroblasts, cementoblasts and osteoblasts [9, 59]. However, stimulating the PDLCs to form anatomically organized tissue is challenging, and a multilayering approach has been recommended. When the PDLC sheet was incubated in the presence of osteogenic induction reagents, the subcutaneous implantation of this cell sheet formed a cementum-like complex in the center of the sheet and PDL tissue properties at the periphery [60]. This study showed the self-assembling properties of PDLCs in the presence of suitable induction molecules.

In another study, three-layered PDL cell sheets showed thick cementum formation in a rat and canine model [6, 9], while monolayer PDL sheet with hyaluronic acid carrier had a limitation on new cementum, PDL and bone formation in a canine model [8].

## Challenges and pathways of cell-based PDL regeneration for avulsed teeth to clinic

### cGMP facility

A commonly known key obstacle for cell-based therapy is when cells have to undergo extensive culturing steps. cGMP facility and FDA approval have to be in place prior to a routine practice for such a clinical condition. Sterile and clinical-grade cells validated by the approved protocols are one essential step for cell-based therapy [61]. The expansion and characterization of PDL cells are important for clinical trials. Iwata et al. [62] reported a successful protocol for the characterization of PDL cells from 41 extracted human teeth. PDL cells showed rapid proliferation at low cell density, differentiated into cementoblasts and osteoblasts, expressed neural cell adhesion molecule 1 (NCAM1), calcium-binding protein (S100A4) and periostin. Thus, they possess the feasibility for a standard protocol of cell culturing and expansion.

### Autologous cell source

Using autologous cells faces several challenges: i) the availability of cell source from the host at the time of need; ii) time needed to culture the cells before they are ready for transplant; iii) the quality of the cells that varies among individuals.

#### PDL cells

Accumulated evidence appears to indicate that PDLSCs may be the best option for this purpose. PDL progenitors were shown to have a higher periostin expression, osteogenic potential and cellular elongation than dental pulp and dental follicle cells [27]. However, if there is a lack of available PDL from the host when needed, this option is not viable. One exception is if the host has preserved his/her own PDL or PDLSCs via a cryopreserving service previously. A study showed cryopreservation of human teeth up to 1 year using a magnetic-programmed freezer and reported that PDL was intact and PDLCs proliferated as much as those from freshly extracted tooth [63]. The same group investigated the effect of cryopreservation in vivo using alveolar sockets of Wistar rats and reported that there was no difference in collagen and ALP synthesis between the immediately replanted teeth group and cryopreserved group, while significant root resorption and ankylosis was observed in dried teeth [64]. On the other hand, the quality of multipotency, self-renewal ability and osteogenic properties of PDL cells decrease with age [65, 66]. Interestingly, Zheng et al. [67] showed that aged PDLSCs (obtained from patients aged 54 ± 3.2 yrs) improved the tissue regeneration capacity to produce cementum-periodontal-like tissues after induction with the conditioned medium from young PDLSCs (obtained from patients aged 15 ± 2.4 yrs) in an ectopic mice model, while aged PDLSCs without any induction presented fibrous tissue formation. This study shows the importance of ECM proteins and the local microenvironment for regenerative properties of PDLSCs.

#### Non-PDL cells

Besides PDL cells, a previous study reported cementum regeneration, the organization of PDL fibers, and bone morphogenetic protein-2 and osteopontin expression after implantation of adipose stromal cells into the first lower molar periodontium of the mice after 12 weeks [68]. Their autologous nature, easy handling, multipotent and high yield properties can be considered advantageous [69]. In another study, the local injection of bone marrow mesenchymal stem cells (BMMSCs) into the periodontal defect in a rat model induced periodontal ligament and bone regeneration with decreased probing depth in the regenerated area (1.2 mm) compared to the untreated area (1.7 mm) [70]. On the other hand, a meta-analysis reported that PDLCs are still superior to BMMSCs for PDL formation [71]. As another potential cell source, induced pluripotent stem cells (iPSCs) have great potential to differentiate into various cell lineages [72, 73]. iPSCs can be reprogrammed from dental-derived cells such as gingival fibroblasts or periodontal ligament fibroblasts by certain factors [74]. In a previous study, the use of iPSCs and BMP-6 in a hydrogel scaffold resulted in periodontal, cementum and bone-like tissue formation compared to a cell-free group in which connective tissue and bone formation occurred [73]. In addition, owing to the immunomodulatory properties of the stem cells, these studies showed less proinflammatory cytokine production and inflammation in the cell-based groups [70, 73].

### Allogeneic cell source

Although the safety and efficacy of autologous PDL-derived cells and dental pulp stem cells for the treatment of periodontal defects have been reported in previous clinical trials, the isolation, characterization and preparation of the autologous cell sheets require 2–3 months before transplantation [75]. An alternative is to use allogeneic PDLSCs which could be cryopreserved and later thawed for timely use. They can be pre-selected for their quality, and genotyping is determined for the best match for the recipient. Like other MSCs, PDLSCs possess immunosuppressive capacity, thereby having low immunogenicity and immunological response, such that cells can sustain in the host after transplantation [36]. PDLSCs maintain low immunogenicity and T cell suppression after osteogenic differentiation [76]. Allogeneic stem cell-based bio-root can be regenerated or repairing periodontal defect due to periodontitis in a swine model without signs of immunologic rejection [36, 77].

Moreover, if allogeneic PDL cells are not available, allogeneic AdSCs or BMMSCs may be an alternative. The immunologic and regenerative properties of allogeneic adipose-derived multi-lineage progenitor cells were investigated in a mini-pig periodontal defect model and showed comparable regeneration ability and higher immune-suppressive cytokine expressions compared to autologous transplantation [78]. Du et al. also used allogeneic BMMSC transplantation for periodontal regeneration in a rat model and reported PDL regeneration and decrease in proinflammatory cytokine expression [70].

### Potential indication for cell-free approach

Certain clinical conditions may allow a cell-free approach to succeed in the regeneration of PDL for avulsed teeth. PDL tissue remains can be observed in the tooth socket 2 weeks after extraction in a human study [79]. Such PDL can provide a cell source for regeneration [80, 81]. Previous studies showed that PDL cells on the extracted tooth socket wall can preserve vitality and migration ability during the first week of healing after the extraction [82, 83].

Mechanical injury to bone can stimulate osteoblasts for bone formation that can interfere with the PDL regeneration since the cell types after injury determine the type of the newly formed tissue. Iwata et al. [84] described a culturing method before transplantation by culturing the extracted tooth in a culture medium for 1 month for the proliferation of the remaining PDL cells that was covered half of the root surfaces. However, during this time, the preservation of the alveolar socket remains challenging for the subsequent tooth replantation.

Under such a condition, besides remaining PDL in the socket can help regeneration, an additional approach can enhance overall regeneration by providing rich growth factors. These growth factors not only help tissue heal but also induce cell homing from the surrounding healthy tissues, mobilizing neighboring stem cells or tissue cells to the PDL space to facilitate regeneration.

Ji et al. [44] tested tooth root treated with EDTA to expose growth factors and replanted into the fresh tooth socket along with PRF, and found that such combined treatment allowed PDL regeneration. However, when replanting such constructs into a socket that has healed for three months and recreated by drilling, the regeneration was unsuccessful and ankylosis occurred. Another study showed that in the absence of PDL cells on the root surface, the conditioning of the root surface with citric acid failed to form PDL and/or cementum formation but resulted in inflammation and epithelial growth rather than new PDL tissue formation in a beagle dog model [85].

In the study by Zhao et al. [10], PRF used alone without PDLSCs did not show the same adequate fiber orientation when PDLSCs were used. Only a very thin layer of cementum or no new cementum was formed in the cell free-PRF group compared to the groups using PDLSCs. Thus, a cell-free approach has its limit and dependent largely on the clinical condition.

Yang et al. [86] applied platelet-rich plasma (PRP) on the curated root surface of the premolars with an external dry time of 5 min, and reported 92% of newly formed PDL-like soft tissue, 51.6% of cementum-like hard tissue and 7.9% of root resorptive areas compared to the saline control which showed a high incidence of resorptive areas (78.3%) and minimal PDL and cement-like tissue formation. This study showed that the application of PRP onto root surface during tooth replantation can reduce tooth ankylosis and increase periodontal ligament- and cementum-like tissue formation.

## Prospective new clinical protocol for management of avulsed teeth

**Fig. 4:**
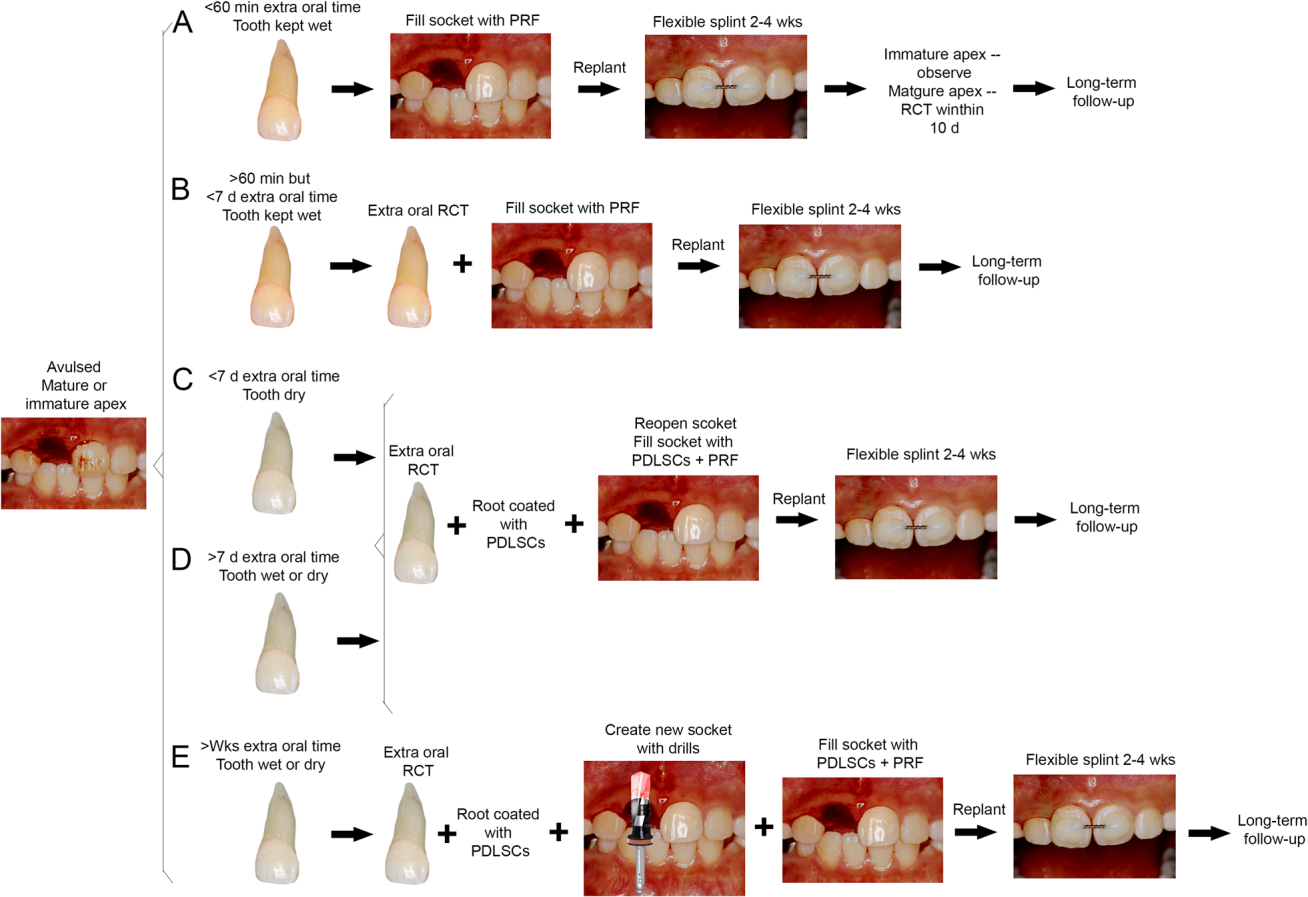
Proposed protocol for the management of avulsed teeth of various conditions. **A**, **B**) Non cell-based PDL regeneration. Avulsed tooth was kept wet in a storage medium. For condition in **B**, the regeneration of PDL relies mainly on the remaining PDL cells in the tooth socket. Addition of PRF is necessary to increase chances of better outcomes. **C**–**E** Cell-based PDL regeneration. Once the avulsed tooth is dried or stayed extra oral time more than 7 days, all cells on the root become non-viable or mostly non-viable. PDL cells in the tooth socket also have reduced or disappeared. Thus, cell-based PDL regeneration is necessary. For condition in **E**, recreating the tooth socket with implant drills is needed because bone has begun to regenerate. Clinical images, courtesy of Dr. Mitsuhiro Tsukiboshi

Based on those key cell-based studies reviewed above as well as the established principle to manage avulsed teeth (https://www.iadt-dentaltrauma.org) [87], we propose a new clinical protocol extending from the current management protocol as to how the avulsed tooth may be handled under various conditions (Fig. 4). We categorized five situations: (A) Extraoral time is less than 1 h and the tooth is kept wet. Immediate replantation along with PRF is advised. (B) Extraoral time is more than 2 h but less than 7 days and the tooth is kept wet. Root canal treatment (RCT) should be done followed by replantation with PRF. The outcome of this situation is certainly less favorable than that in (A) because the PDL regeneration is heavily relying on the remaining PDL in the tooth socket. Although an afore-mentioned study found that PDL remains are present after 2 weeks of extraction, it is uncertain whether this allows optimal PDL regeneration after replantation. A case report showed that a dry tooth was replanted 6 days after avulsion survived at 1-year followed up with mild ankylosis [88]. Another case showed replacement resorption at 1-year recall even though the tooth was kept wet (milk) and replanted the next day [89]. (C) Extraoral time is less than 7 days but the tooth is dry. RCT followed by cell-mediated therapy is the only optimal way. (D) Extraoral time is more than 7 days either tooth is kept wet or dry; RCT followed by cell-based therapy is the only option. (E) Extraoral time is longer than weeks, either kept wet or dry. RCT is followed by creating socket space, which by then has been filled with granulation tissue or immature bone, for the cell-based replantation procedures. If the cell-based therapy is well established, category (B) situation may consider cell-based approach because its outcome is heavily dependent on what kind of wet condition the tooth is kept and how much PDL is still remaining in the tooth socket.

## Prospect and conclusions

The progress of regeneration research, especially cell therapy, has provided much possibility to overcome the limitations and challenges that non cell-based therapy has faced in many medical conditions. To advance the treatment of avulsed teeth, especially those that the PDL has been dry and severely damaged/lost, we must take advantage of what stem cell-mediated approach can provide [41]. Without taking such a leap, management of avulsed teeth will continue to be stagnated.

For cell-based approaches, allogeneic PDLSCs are clinically more practical with the premise that clinical-grade PDLSCs are available in a cell banking system. Autologous PDLSCs, although not impossible, are less likely to be practical for such management. PRF from the host is easily accessible and should be utilized. Besides the issue of a cGMP facility, which is discussed above, another area that requires intensive investigation is the coating of PDLSCs onto the root. Lee et al. [12] so far were the only recent group focusing on the concern of cell attachment onto the root before transplantation. Their experiment had the tooth outside for 5 days and received various treatments, including sterilization. This concern is to identify an optimal way to manage avulsed teeth that have been inappropriately stored or handled such as dried or contaminated which is likely a frequent situation.

From our experiment reported above, cell attachment well onto the root surface before replantation appears to be one of the critical factors for successful PDL regeneration. Cell sheet technique is technically sensitive, and a more standardized protocol is required for a predictable outcome. An optimal and reliable method to seed the cells onto the root surface that has a highly variable and irregular topography needs to be developed, for example 3D bioprinting, a technology that uses live cells and biocompatible materials to manufacture a tissue-like structure. With the flexibility and precision of this technology, we may seed multiple cell types and growth factors in any combinations we deem optimal.

Maintaining the viability of stem cells during the attachment and surgery process is an important aspect to be carefully studied. If the avulsed tooth is dry or extraoral time is longer than 7 days with a healed alveolar socket, an edentulous approach can be used as described in the current study using implant drills. However, this approach resulted in inflammation and resorption on the root surface by the replantation procedure before PDL tissue regeneration. This finding showed that PDL regeneration in the presence of immunomodulatory approaches is required to improve the predictability of the outcome.

Since the microenvironmental cues are essential for directing stem cell fate in vivo, it is critical to provide an adequate condition to guide the PDLSCs into cementoblast lineages and prevent unwanted differentiation before implantation. Understanding the role of ECM proteins and their functions is also vital for achieving predictable tissue regeneration. The use of cementogenic induction agents such as ascorbic acid seems beneficial for ECM synthesis and PDL and cementum regeneration [47].

Any new technology or approach that can ensure a suitable cell attachment onto the root surface and, simultaneously, guide the cells toward cementum and PDL generating cells for timely tissue regeneration in the presence of immunomodulation as soon as the tooth is replanted appears critical for a long-term successful outcome.

## Supplementary Information

**Additional file 1: Fig. S1**. In vitro preparation of hPDLSCs cell sheet from cells grown in UpCell^tm^ temperature-responsive dish. (**A**) Three layers of PDLSC-sheet using UpCell^tm^ temperature-responsive dish and attached onto PGA membrane. After placing the dish at room temperature, the membrane (a) is placed on the first layer of cells (b) after attachment of the cells to the membrane, the membrane and the first layer are placed over the second layer of cells (c) in another dish. The same step is repeated again for the third layer of cells (d). (**B**) PGA/PDLSC-sheet wrapped onto root fragment and tied with resorbable sutures. Membrane (a) attached to the cell sheet (b) is adapted and wrapped around a prepared root (c). The membrane/cell sheet is tied with Vicryl 5-0 (d). The cell sheet is facing the root surface and the membrane is on the outside.

**Additional file 2: Fig. S2**. Formation of cementum-like and PDL-like tissue in a mouse model. (**A**) Swine PDLSCs were examined for their multipotent differentiation capacity. Odontogenic (Odon, 5 weeks of stimulus); Ad: adipogenic; and Neuro: neurogenic. Ctrl: control group without differentiation stimulation. (**B**) Swine or human PDLSCs mixed with HA/TCP and transplanted into SCID mice subQ for 2-3 months and then processed for histology. C: cementum-like. Blue arrows: Sharpey’s fiber-like or collagen bundles in PDL-like tissues. Scale bar: (A) Top/middle panels: 200 µm; bottom panel: 50 µm; (B) top panel: 500 µm, middle: 100 µm; bottom: 50 µm. Differentiation and staining protocols followed studies published previously [47, 48].

**Additional file 3: Fig. S3**. Orthotopic PDL regeneration for swine teeth. (**A**) Allogeneic swine PDLSC-sheet wrapped root fragment; (**B**) insertion of root fragment into an tooth extraction site; (**C**) root inserted deep into the socket and wound sutured; (**D**) radiograph showing inserted root below alveolar crest line; (**E**) 6 moths at the sacrifice, root fragment extruded.

## Data Availability

All data generated or analyzed during this study are included in this published article [and its Additional files 1–3].

## Abbreviations

AdSCs: Adipose tissue-derived stem cells
GMSC: Gingival mesenchymal stem cell
HA/TCP: Hydroxyapatite/tricalcium phosphate
HBSS: Hanks' balanced salt solution
MMPs: Matrix metalloproteinases
NCAM1: Cell adhesion molecule 1
PDL: Periodontal ligament
PDLPs: Periodontal ligament progenitor cells
PDLSCs: Periodontal ligament stem cells
PGA: Polyglycolic acid
PIPAAm: Poly N-isopropylacrylamide
PRF: Platelet-rich fibrin
PRP: Platelet-rich plasma
RCT: Root canal treatment
