# Supplementary figures and images for: A new direction in managing avulsed teeth: stem cell-based de novo PDL regeneration
Source: Stem Cell Res Ther. 2022 Jan 28;13:34. doi: 10.1186/s13287-022-02700-x (PMC8796335; doi:10.1186/s13287-022-02700-x)

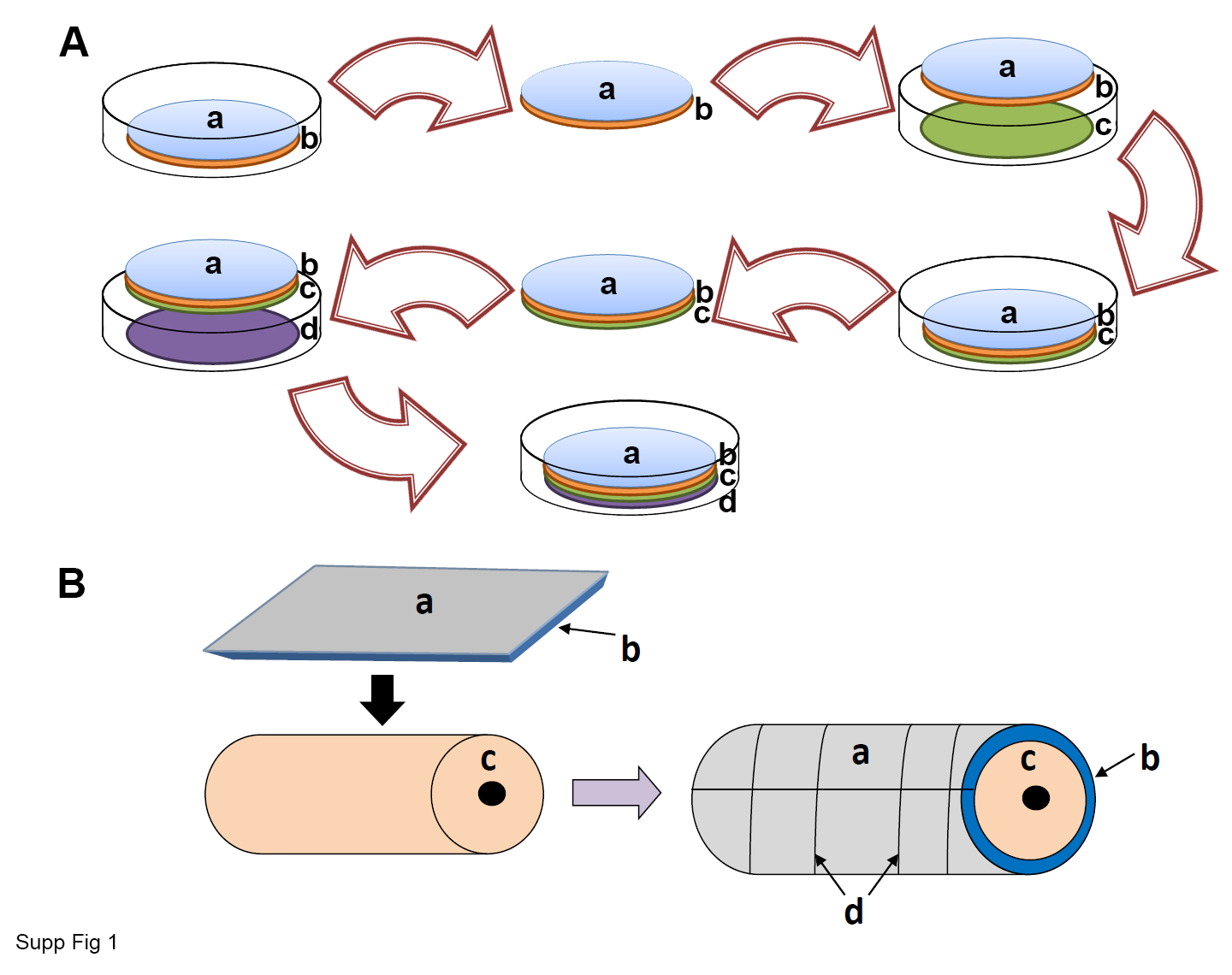

Supplement: Supplementary file 1 — Additional file 1: Fig. S1. In vitro preparation of hPDLSCs cell sheet from cells grown in UpCelltm temperature-responsive dish. (A) Three layers of PDLSC-sheet using UpCelltm temperature-responsive dish and attached onto PGA membrane. After placing the dish at room temperature, the membrane (a) is placed on the first layer of cells (b) after attachment of the cells to the membrane, the membrane and the first layer are placed over the second layer of cells (c) in another dish. The same step is repeated again for the third layer of cells (d). (B) PGA/PDLSC-sheet wrapped onto root fragment and tied with resorbable sutures. Membrane (a) attached to the cell sheet (b) is adapted and wrapped around a prepared root (c). The membrane/cell sheet is tied with Vicryl 5-0 (d). The cell sheet is facing the root surface and the membrane is on the outside. [file 13287_2022_2700_MOESM1_ESM.tif]

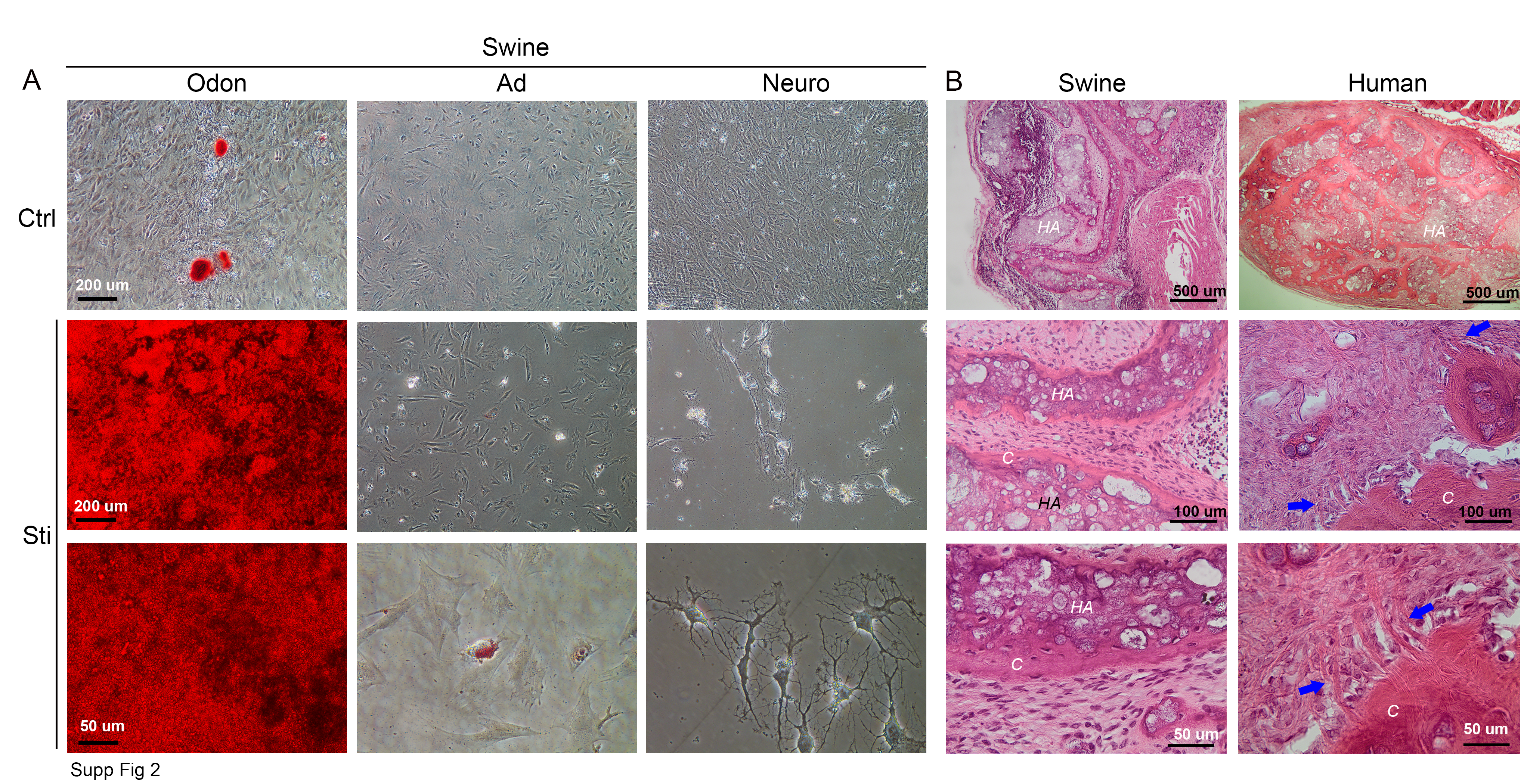

Supplement: Supplementary file 2 — Additional file 2: Fig. S2. Formation of cementum-like and PDL-like tissue in a mouse model. (A) Swine PDLSCs were examined for their multipotent differentiation capacity. Odontogenic (Odon, 5 weeks of stimulus); Ad: adipogenic; and Neuro: neurogenic. Ctrl: control group without differentiation stimulation. (B) Swine or human PDLSCs mixed with HA/TCP and transplanted into SCID mice subQ for 2-3 months and then processed for histology. C: cementum-like. Blue arrows: Sharpey’s fiber-like or collagen bundles in PDL-like tissues. Scale bar: (A) Top/middle panels: 200 µm; bottom panel: 50 µm; (B) top panel: 500 µm, middle: 100 µm; bottom: 50 µm. Differentiation and staining protocols followed studies published previously [47, 48]. [file 13287_2022_2700_MOESM2_ESM.tif]

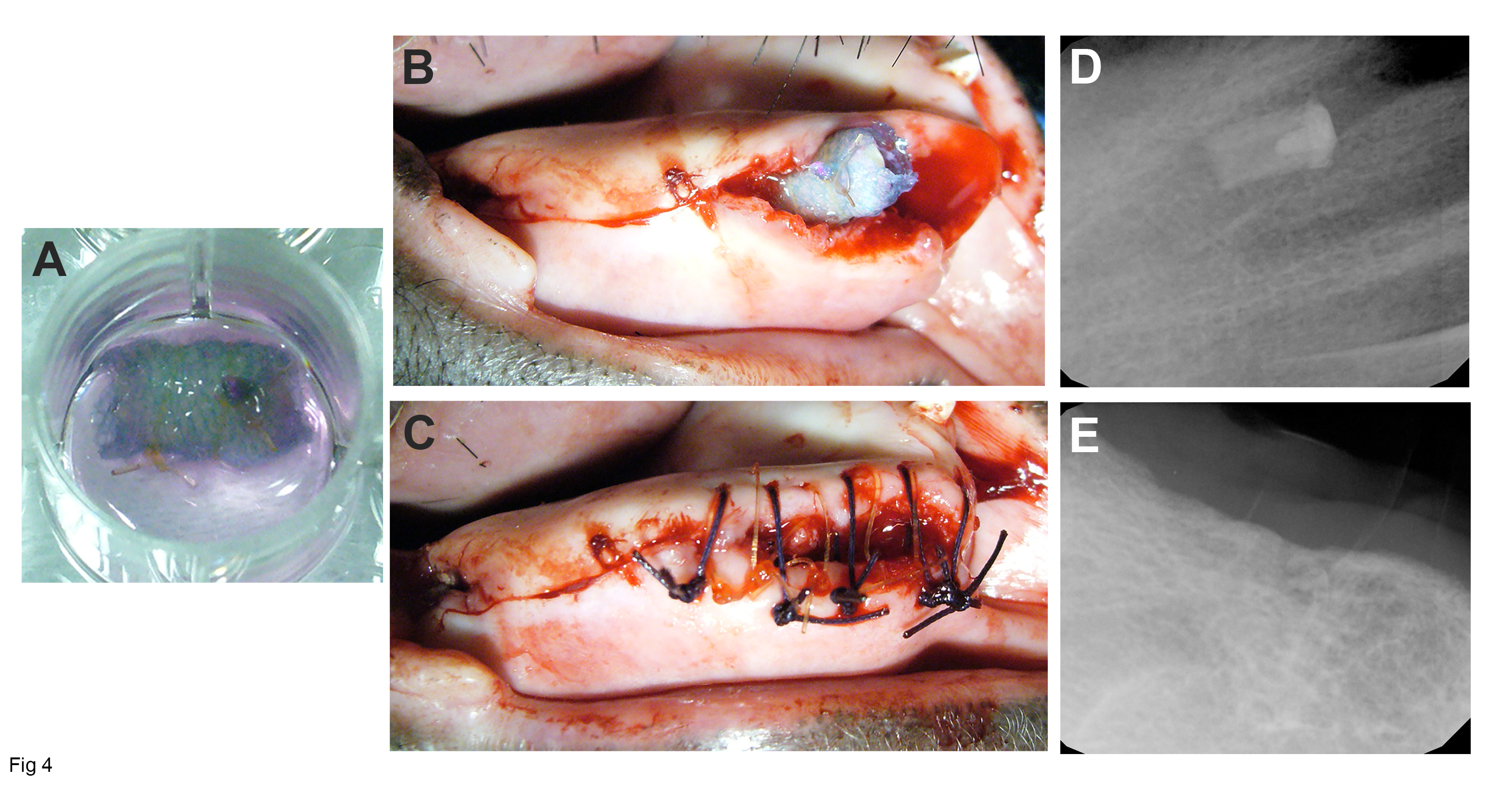

Supplement: Supplementary file 3 — Additional file 3: Fig. S3. Orthotopic PDL regeneration for swine teeth. (A) Allogeneic swine PDLSC-sheet wrapped root fragment; (B) insertion of root fragment into an tooth extraction site; (C) root inserted deep into the socket and wound sutured; (D) radiograph showing inserted root below alveolar crest line; (E) 6 moths at the sacrifice, root fragment extruded. [file 13287_2022_2700_MOESM3_ESM.tif]
